# Primary atypical teratoid/rhabdoid tumor of the optic nerve: a rare entity in an exceptional location

**DOI:** 10.1186/s13000-015-0284-2

**Published:** 2015-05-02

**Authors:** Youssef Mahdi, Jinane Kharmoum, Amal Alouan, Hakima Elouarradi, Iman Elkhiyat, Mustapha Maher, Moulay Zahid Benchrif, Amina Kili, Rajae Daoudi, Nadia Cherradi

**Affiliations:** Department of Pathology, Specialities Hospital, Ibn Sina University Hospital, Rabat, Morocco; Faculty of Medicine and Pharmacy, Mohammed V Souissi University, Rabat, Morocco; Department of Ophtamology, Specialities Hospital, Ibn Sina University Hospital, Rabat, Morocco; Pediatric Hematology and Oncology Center, Ibn Sina University Hospital, Rabat, Morocco

**Keywords:** Rhabdoid, Atypical teratoid, Optic nerve

## Abstract

**ᅟ:**

Atypical teratoid/rhabdoid tumors are rare and highly malignant central nervous system tumors. They have no specific radiological features and often present several histological components that make a problem in differential diagnosis with medulloblastoma and primitive neuroectodermal tumors.

We present the case of a newborn girl complained of a gradual proptosis of the left eye secondary to an expansive lesional process of the optic nerve. The location at the optic nerve, reported only twice in the literature, and an exclusive rhabdoid appearance on biopsy added additional differential diagnosis problems.

The proptosis worsened and the infant died few days after two cycles of chemotherapy.

**Virtual slides:**

The virtual slides for this article can be found here: http://www.diagnosticpathology.diagnomx.eu/vs/2037718783145212.

## Background

World Health Organization (WHO) defines atypical teratoid/rhabdoid tumour (AT/RT) as a highly malignant central nervous system (CNS) tumour predominantly manifesting in young children, typically containing rhabdoid cells, often with primitive neuroectodermal cells and with divergent differentiation along epithelial, mesenchymal, neuronal or glial lines; associated with inactivation of the integrase interactor (INI)1/hSNF5 gene in virtually all cases [1]. AT/RTs have non-specific clinical and radiological features, often present several components at histology and are commonly infratentorial, making a problem in differential diagnosis with medulloblastoma, primitive neuroectodermal tumors (PNET) and germ cells tumors. In our case, the location at the optic nerve, reported only twice in the literature, added an additional differential diagnosis problem with optic nerve gliomas. Furthermore, exclusive rhabdoid appearance on biopsy referred us first to rhabdomyosarcoma.

## Case presentation

### Clinical history

A newborn girl, without perinatal history, presented at birth a gradual increase of the left eye volume. At day 20 of life, parents decided to consult a doctor. Physical examination showed proptosis of the left eye.

### Radiologic and histopathologic findings

**Figure 1:**
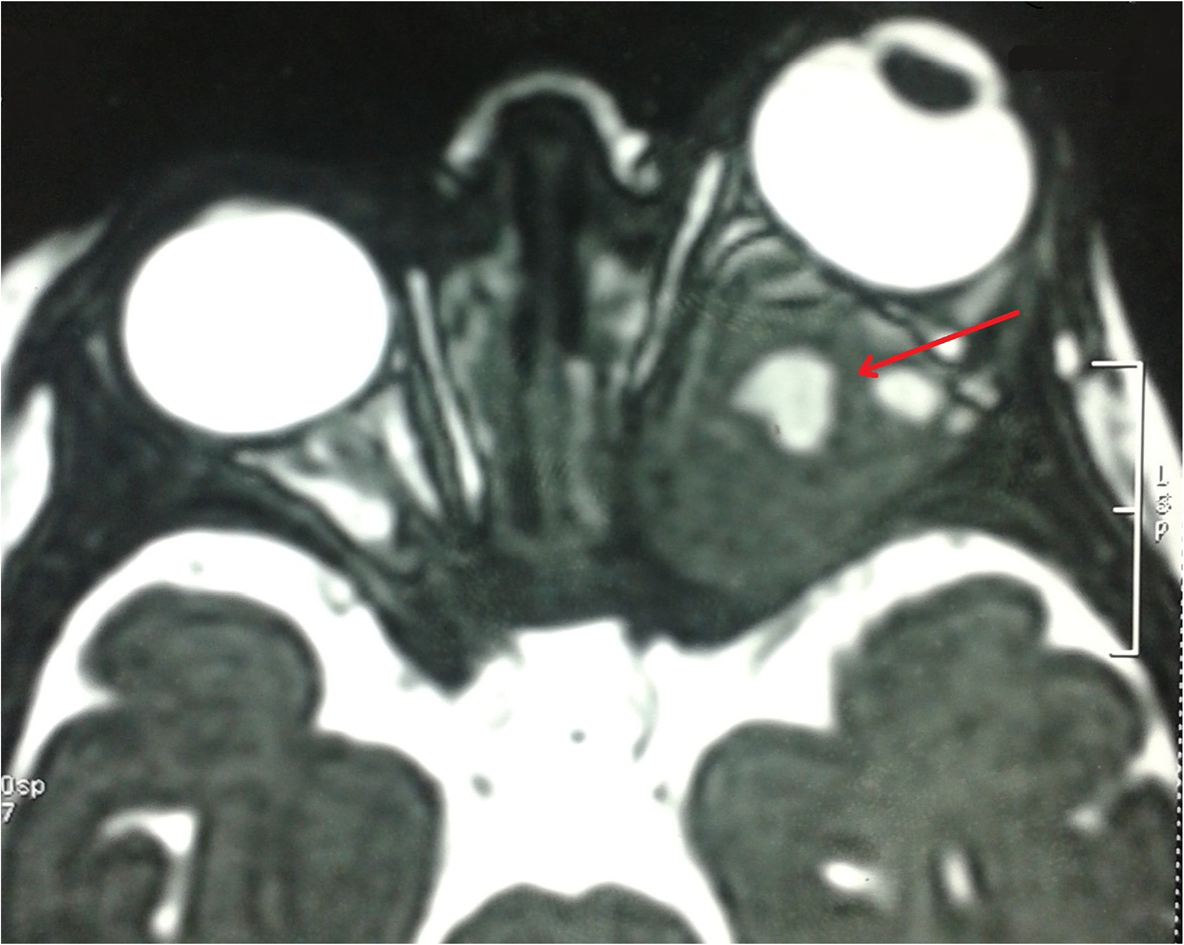
MRI aspect in admission. Axial T2-weighted MR image shows hyperintense signal and enlargement of the left optic nerve (arrow).

**Figure 2:**
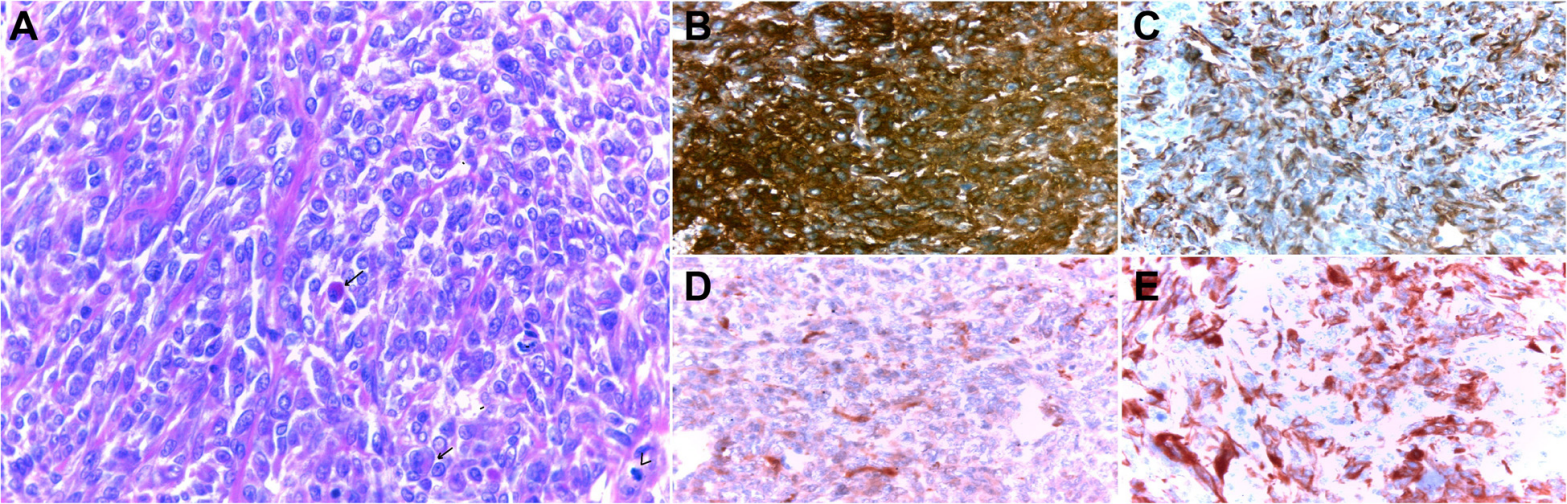
Histological aspect and immunohistochemical profile of the lesion. **(A)**. The cytoplasm of neoplastic cells contains sometimes eosinophilic globular inclusions (arrows). The nucleus is irregular with vesicular chromatin and several mitotic figures (arrowhead) (H & E × 400). The tumor cells are positive for EMA **(B)**, vimentin **(C)**, S100 protein **(D)** and pancytokeratin **(E)**.

**Figure 3:**
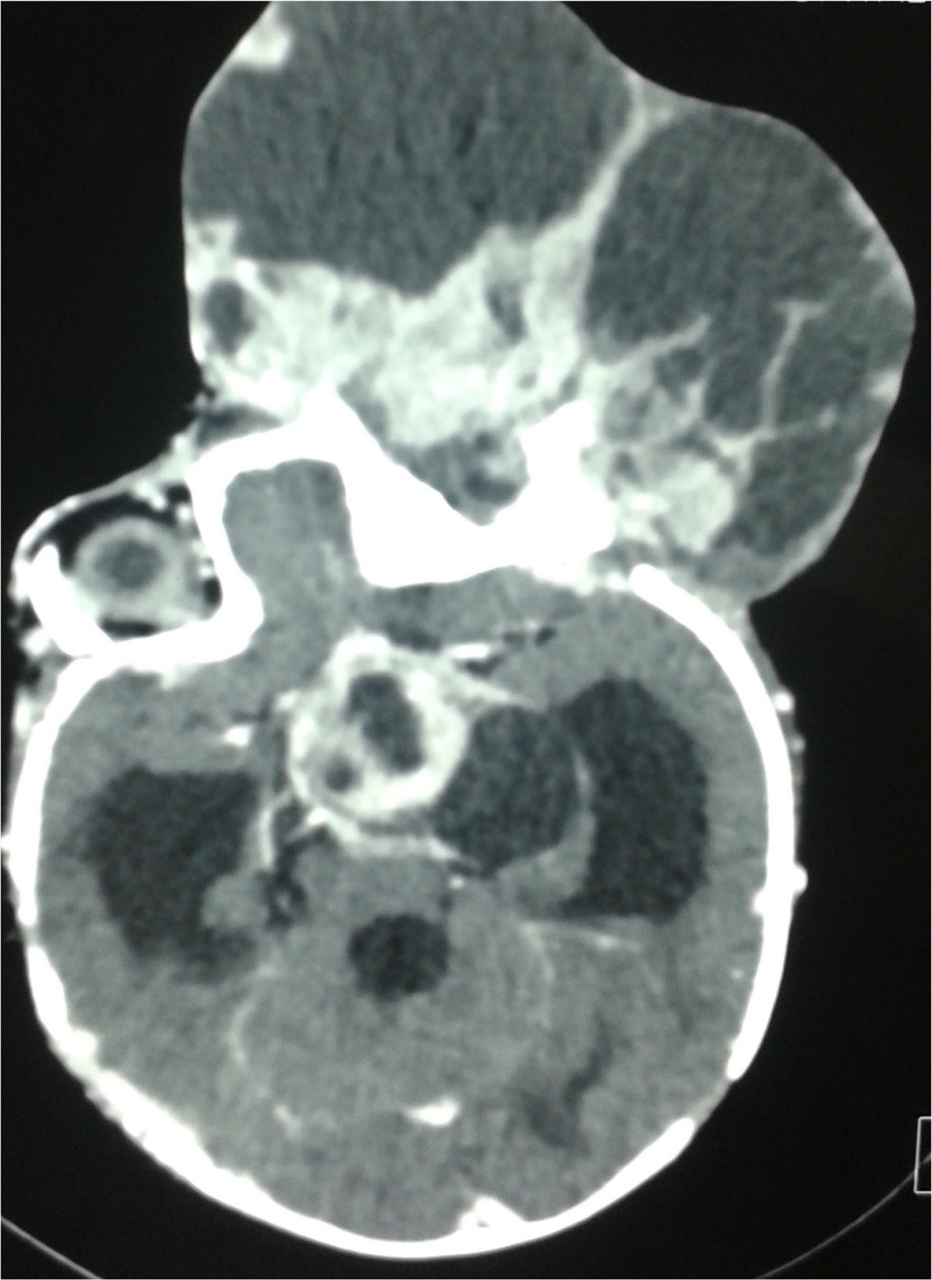
Evolution after 2 courses of chemotherapy. Axial T2-weighted MR image redone objectives a huge heterogeneous orbito-cerebral mass, enhanced intensely after injection of contrast agent and associated to triventricular dilatation.

The ultrasound revealed 2 cm hypoechoic formation occupied the posterior pole of the orbit without Doppler vascularity, well limited by printing capsule at the bottom and outside of the optic nerve. The globe was normal. Orbital magnetic resonance imaging (MRI) revealed an expansive fusiform lesional process in intraconal space encompassing the optic nerve, measured 25/19/17 mm (Figure 1). This aspect evoked optic nerve glioma. Bilateral cortical atrophy in frontotemporal cortex was also observed. Thoraco-abdominal computed tomography (CT) showed right renal malrotation without tumor. As a result, a biopsy was performed. Microscopically, we observed sheets of round and sometimes slightly fusiform neoplastic cells. Their cytoplasm was eosinophilic and sometimes contained eosinophilic globular inclusions. The nucleus was irregular, with vesicular chromatin and several mitotic figures estimated at 8 mitoses per 10 high power fields (Figure 2A). The immunohistochemical study showed reactivity for epithelial membrane antigen (EMA) (Figure 2B), vimentin (Figure 2C), S100 protein (Figure 2D), pancytokeratin (Figure 2E) and glial fibrillary acidic protein (GFAP). The tumor was immunonegative for desmin, myogenin, INI1, chromogranin, synaptophysin, CD34, CD99 and CK5/6. The diagnosis was atypical teratoid/rhabdoid tumor. The patient received cyclophosphamide/vincristine/ifosfamide/actinomycine-based chemotherapy. Evolution was marked by a worsening of proptosis after two cycles of chemotherapy. A new MRI redone objectified a huge heterogeneous left orbito-cerebral mass contained calcifications, enhanced intensely after injection of contrast agent, measured 110 and 100 mm (Figure 3). It showed a bone lysis of the orbital frame, invasion of the left cavernous sinus, extension and infiltration of the temporal lobe and supra sellar region. Lesion resulted of mass effect on the lateral ventricles causing triventricular dilatation. The infant died few days after second course of chemotherapy.

### Discussion

Malignant rhabdoid tumors were originally described in kidney then at other sites especially soft tissue [2-9]. In 1985, Briner et al. reported the first case of primary cerebral rhabdoid tumor [10]. The term AT/RT was proposed for this entity by Rorke et al. because of the combination of rhabdoid, primitive neuroepithelial, epithelial and mesenchymal components [11]. AT/RTs represent 1–2% of paediatric brain tumours [1]. They usually occur in children under the age of 3 years, and rarely affect those older than 6 years, with a median age of 2 years [1]. There is a male predominance ranging from 1.6–2:1 [1]. Presenting symptoms are non-specific, represented by lethargy, vomiting or failure to thrive in infants, headache and hemiplegia in children older than three years [1].

AT/RTs are most often infratentorial [12,13], located in the cerebellar hemispheres, cerebellopontine angle or brain stem [1,12]. Supratentorial tumors are commonly located in the cerebral hemispheres, and less frequently in the ventricular system, suprasellar region or pineal gland [1]. They rarely arise in the spinal cord [1,13]. Seeding of AT/RTs via the cerebrospinal fluid pathways is found in more than 20% of the patients at presentation [1].

AT/RT of the optic nerve, as in our case, is exceptional and only two cases were reported in the literature. In 2006, Allen et al. described a case of an AT/RT of the optic nerve secondarily arising in a ganglioglioma [14]. Verma et al. reported in 2008 a case of primary AT/RT in optic nerve in 2-years-old boy [15]. In addition, Fujita et al. reported in 2005 a case of multicentric AT/RTs occurring in the eye and fourth ventricle but without involvement of optic nerve [16].

CT reveals a hyperdense solid component with moderate to marked enhancement with contrast medium [17]. MRI is the recommended imaging modality for the complete evaluation of the optic pathway [18]. It often shows isointense or hypointense signal on T1 and T2 weighted images [17]. There is a tendency for calcification, hemorrhage, necrosis and perifocal edema [17]. At the optic nerve, these radiological features are similar to optic nerve gliomas (ONG), accounting for 66% of all primary tumors of the optic nerve [18]. However, ONG are hyperintense to cerebral cortex on the T2-weighted images [18]. In addition, the peak age of occurrence ranges from 2 to 8 years old and typically follows an indolent course [18]. In another sites, the imaging findings are similar to those seen in patients with medulloblastoma or PNET. Compared with medulloblatomas, AT/RTs have an increased rate of cerebellopontine angle involvement and intratumoral hemorrhage [19].

Macroscopically, AT/RTs tend to be soft, pinkish-red and bulky, and typically contain necrotic foci and may be haemorrhagic [1].

Histologically, the most striking feature are nests or sheets of cells with classic rhabdoid features: abundant cytoplasm contains paranuclear eosinophilic globular inclusion, eccentrically placed nuclei with a vesicular chromatin and a prominent nucleoli [1,12]. By electron microscopy the cytoplasmic inclusions correspond to bundles of intermediate filaments forming tight whorls [12]. The rhabdoid cells can have less striking nuclear atypia and large amounts of pale eosinophilic cytoplasm [1]. However, cases in which the cells are the exclusive or predominant are rare, and most tumours contain variable components with primitive neuroectodermal, mesenchymal and epithelial features [1]. Primitive neuroectodermal component is encountered in two thirds of cases and have the appearance of other small blue cell tumors of childhood [1,12]. Mesenchymal and epithelial differentiations are less common and appear as areas with spindle cell features in the first one and as papillary structures, adenomatous areas or poorly differentiated ribbons and cords in the second one [1]. In immunohistochemical study, AT/RTs display a polyphenotypic immunophenotype [1,12]. However, the rhabdoid cells characteristically demonstrate consistent expression of epithelial membrane antigen and vimentin [1]. Expression of smooth muscle actin, GFAP, neurofilament protein, synaptophysin and keratins are also commonly observed [1,12]. Loss of nuclear expression of INI1 in tumour cells has been shown to be a sensitive and specific marker for AT/RTs [1,20]. It may have particular utility in case of tumors with indeterminate histologic features or atypical immunophenotypic profiles [20].

In our case, we dealt with a biopsy showed only rhabdoid cells. The problem of differential diagnosis arose with rhabdomyosarcoma. The negativity of desmin at first then the negativity of the INI 1 allowed us to rectify the diagnosis. In other locations, the differential diagnosis includes medulloblastomas, PNET and germ cells tumors. ATRT shares some morphological features with those entities, but the presence of rhabdoid cells as well as immunohistochemical study gave the correct diagnosis.

Deletions and mutations of the hSNF5/INI1/SMARCB1 locus at 22q11.2 were demonstrated in AT/RTs as well as in rhabdoid tumors of the kidney and extra-renal sites [21,22]. It suggested that INI1 is a tumor suppressor gene involved in rhabdoid tumors [22]. AT/RTs can occur sporadically or as part of rhabdoid tumor predisposition syndrome [1].

For treatment, there are no definitive guidelines for AT/RTs because of their rarity. However, it based on surgical resection, chemotherapy and radiotherapy. Surgical resection must be as radical as possible. Indeed, patients undergoing gross total resection have a better survival compared to the patients with incomplete resection [13,23]. Radiotherapy is crucial in the treatment and can be focal or craniospinal [23]. It recommended to initiate it immediately postoperatively and before systemic chemotherapy in pediatric patients older than 3 years [23]. For chemotherapy, several drugs are used in various regimens including: vincristine, carboplatin, cisplatinum, cyclophosphamide, etoposide, ifosfamide, methotrexate, lomustine and temozolomide [13]. High dose chemotherapy regimens are associated with a significant survival benefit [13]. However, the used of intrathecal chemotherapy is not associated with a significant prognostic value [13].

AT/RTs correspond to WHO grade IV, having a poor prognosis [1]. The mean survival is 6–11 months [12].

## Conclusion

In summary, AT/RTs are very aggressive CNS embryonal tumors that make several problems in differential diagnosis. In our case, the location and the histological aspect posed additional diagnostic difficulties. An accurate diagnosis is imperative because AT/RTs have worse prognosis. At that context, pathological analysis is critical.

## Consent

Written informed consent was obtained from the parents of the patient for publication of this Case Report and any accompanying images. A copy of the written consent is available for review by the Editor-in-Chief of this journal.

## Abbreviations

WHO: World Health Organization
AT/RT: Atypical teratoid/rhabdoid tumour
CNS: Central nervous system
INI: Integrase interactor
PNET: Primitive neuroectodermal tumors
MRI: Magnetic resonance imaging
CT: Computed tomography
EMA: Epithelial membrane antigen
GFAP: Glial fibrillary acidic protein
ONG: Optic nerve gliomas
